# The Obemat2.0 Study: A Clinical Trial of a Motivational Intervention for Childhood Obesity Treatment

**DOI:** 10.3390/nu11020419

**Published:** 2019-02-16

**Authors:** Veronica Luque, Albert Feliu, Joaquín Escribano, Natalia Ferré, Gemma Flores, Raquel Monné, Desirée Gutiérrez-Marín, Núria Guillen, Judit Muñoz-Hernando, Marta Zaragoza-Jordana, Mariona Gispert-Llauradó, Carme Rubio-Torrents, Mercè Núñez-Roig, Mireia Alcázar, Raimon Ferré, Josep M. Basora, Pablo Hsu, Clara Alegret-Basora, Francesc Arasa, Michelle Venables, Priya Singh, Ricardo Closa-Monasterolo

**Affiliations:** 1 Pediatric Nutrition and Human Development Research Unit, Universitat Rovira i Virgili, IISPV, 43201 Reus, Spain; veronica.luque@urv.cat (V.L.); albert.feliu.rovira@gmail.com (A.F.); joaquin.escribano@urv.cat (J.E.); natalia.ferre@urv.cat (N.F.); raquel.monne@urv.cat (R.M.); desiree.gutierrez@urv.cat (D.G.-M.); nuria.guillen@urv.cat (N.G.); judit.munoz@iispv.cat (J.M.-H.); marta.zaragoza@urv.cat (M.Z.-J.); mariona.gispert@urv.cat (M.G.-L.); carme.rubio@urv.cat (C.R.-T.); merce.nunez@iispv.cat (M.N.-R.); mireia.alcazar@iispv.cat (M.A.); 2 Hospital Universitari de Tarragona Joan XXIII, 43007 Tarragona, Spain; 3 Hospital Universitari Sant Joan de Reus, 43204 Reus, Spain; rferre@grupsagessa.com (R.F.); clara.alegret@grupsagessa.com (C.A.-B.); 4 Unitat de Suport a la Recerca Tarragona-Reus, IDIAPJGol, 43204 Reus, Spain; gemmaflores@gmail.com (G.F.); jbasora.tarte.ics@gencat.cat (J.M.B.); pcsss420@gmail.com (P.H.); 5 Hospital Verge de la Cinta, 43500 Tortosa, Tarragona, Spain; arasafrancesc@gmail.com; 6 MRC Elsie Widdowson laboratory, Cambridge CB1 9NL, UK; Michelle.Venables@mrc-epid.cam.ac.uk (M.V.); priya.ann.singh@gmail.com (P.S.)

**Keywords:** motivational interview, childhood obesity, obesity treatment, primary care

## Abstract

The primary aim of the Obemat2.0 trial was to evaluate the efficacy of a multicomponent motivational program for the treatment of childhood obesity, coordinated between primary care and hospital specialized services, compared to the usual intervention performed in primary care. This was a cluster randomized clinical trial conducted in Spain, with two intervention arms: motivational intervention group vs. usual care group (as control), including 167 participants in each. The motivational intervention consisted of motivational interviewing, educational materials, use of an eHealth physical activity monitor and three group-based sessions. The primary outcome was body mass index (BMI) z score increments before and after the 12 (+3) months of intervention. Secondary outcomes (pre-post intervention) were: adherence to treatment, waist circumference (cm), fat mass index (z score), fat free mass index (z score), total body water (kg), bone mineral density (z score), blood lipids profile, glucose metabolism, and psychosocial problems. Other assessments (pre and post-intervention) were: sociodemographic information, physical activity, sedentary activity, neuropsychological testing, perception of body image, quality of the diet, food frequency consumption and foods available at home. The results of this clinical trial could open a window of opportunity to support professionals at the primary care to treat childhood obesity. The clinicaltrials.gov identifier was NCT02889406.

## 1. Introduction

Childhood obesity treatment is challenging and not always cost-effective. Several interventions and programs to treat childhood obesity have been tested, but recent reviews showed a highly variable efficacy, with slight reductions in body mass index (BMI) [1,2]. 

Generally, treatment consists of promoting changes in lifestyle habits related to diet and physical activity. Although there exist strategies focusing only on physical activity or diet, multicomponent interventions consisting of dietary modification, physical activity, behavioral therapy, and education have shown to improve body mass index (BMI), blood pressure, and lipids profile [3,4]. A Cochrane review published by Mead and collaborators in 2017, reported that multi-component behavior-changing interventions considering diet and physical activity could be effective in achieving small, short-term reductions in BMI in children aged 6 to 11 years [2].

In a study in which health professionals were interviewed to identify the main barriers for effectiveness of the treatment, they considered that the lack of motivation of the adolescent patients as well as the lack of family engagement were the main reasons to not achieve the success of the intervention [5]. In this regard, a review by Poobalan in 2010 concluded that the most significant weight reductions were obtained by combining diet and exercise, but also motivational strategies [6]. 

The motivational interview has been used for achieving behavioral changes in different pathologies [7]. Miller defined it as direct assistance with a strong psychological basis and patient-focus; the aim should be to cause a permanent change in behavior, helping to resolve conflicting feelings about the same thing [8]. Motivation was defined as “brain processes that energize and direct behaviour” [9,10]. In an observational prospective study published by Feliu et al. in 2013 [11], a motivational therapy was highly effective in treating adolescent obesity, achieving a BMI z score reduction of 0.5 standard deviations [11].

There is scientific evidence from motivational strategies other than the motivational interview itself. For example, engaging obese children in group-based sessions may enhance continued adherence and cohesion to a lifestyle intervention program [12]. On the other hand, other motivational methods as the use of eHealth devices to self-monitor physical activity is currently under study. The use of wearable sensor monitors to increase physical activity has been tested in youth, delivering some preliminary data to suggest that these devices may offer the potential to increase activity levels [13,14], however evidence in children is still scarce [15].

Obesity treatment interventions in childhood and adolescence have been carried out in a wide range of settings, community-based, school-based, primary care, specialized in clinical settings interventions or internet-based programs, with varying results. In 2014, Kothandan concluded that interventions at family level were more effective in reducing weight, body mass index (BMI) and waist circumference than those made only by addressing the child or adolescent in the school environment [16]. Recently, the American Academy of Pediatrics outlined several stages of the treatment, from which the first two included the primary care support [17]. According to these statements, primary care providers should offer motivational interviewing to achieve healthy lifestyle modifications in family behaviors or environments, and children requiring the next level of obesity treatment, would benefit from receiving a “structured weight management”, receiving additional support beyond the primary care provider (such as a dietitian, physical therapist, or mental health counsellor) [17].

The Obemat2.0 trial (clinicaltrials.gov identifier NCT02889406) was designed and conducted to implement and to test the efficacy of a structured multicomponent motivational therapy, coordinated between hospital dieticians and primary care providers to treat childhood obesity.

## 2. Study Hypothesis

The introduction of a family orientated multicomponent motivational intervention, with a coordinated approach between reference units specialized in clinics and primary care services from the same health region can be an effective tool for the treatment of childhood obesity.

### 2.2. Objectives

#### 2.2.1. Primary Objective

To evaluate the efficacy of a multicomponent motivational intervention for the treatment of childhood obesity (change in BMI z score), coordinated between primary care and hospital specialized services, integrating motivational individual interviews, educational groups and eHealth tools (wearable), compared to the usual intervention performed in pediatrics.

#### 2.2.2. Specific Objectives

To evaluate the efficacy of a multicomponent motivational intervention compared to usual intervention performed in regular pediatrics practice on

- Reducing BMI z score,
- improving metabolic control (insulin resistance and lipids profile),
- improving body composition (fat mass and lean mass z scores),
- increasing physical activity,
- acquiring a healthy eating pattern,
- reducing psychosocial problems,
- increasing the adherence to the obesity treatment program in children with obesity between 8 and 13 years old.

Secondary to the intervention clinical trial, as observational analyses, this study proposed the following secondary objectives:

- To assess the precision of body composition techniques (air-displacement plethysmography, dual X-ray absorptiometry, and biological impedance) to detect changes in body composition over time in children with obesity and to validate its use against the four component model
- To assess vascular function, by measuring the intima media thickness and estimating blood vessels properties (distension and rigidity), in relation to the obesity degree and metabolic profile
- To assess the respiratory function, and the association between the obesity degree and the degree of bronchial obstruction.

## 3. Materials and Methods

### 3.1. Study Design and Study Setting

Randomized clustered clinical trial, with a treatment on children with obesity lasting 12 (+3) months, with two arms: a control group following the usual recommendations in primary care and an intervention group receiving a structured motivation-based interview supported by educational materials, combined with group therapy and eHealth. The investigators registered the trial at clinicaltrials.gov as NCT03749200.

### 3.2. Context and Time Frame of the Study

The project, conducted in Spain, started in January 2016. Up to March 2016, we designed the structure of the intervention and conducted the first round of recruitment of pediatricians and pediatric nurses at primary care centers. In March 2016, pediatricians and pediatric nurses willing to participate in the study as therapists were trained. The recruitment of children with obesity started in June 2016 and ended in March 2018. The baseline and final assessment of participants were conducted at the reference hospitals of the area—Hospital Universitari de Tarragona Joan XXIII and Hospital Universitari Sant Joan de Reus—and the treatment of the obese participants took place in the primary care centers of the Tarragona region, all of that between June 2016 and June 2019.

### 3.3. Sample Size

We considered the number of individuals of a simple random design, multiplied by the design effect to calculate the sample size of each group. Assuming a standard deviation of 0.75 and a 30% lost to follow up, 98 children with obesity were required in each group to detect a difference ≥ 0.36 units of BMI z score (with and alpha risk = 0.05 and beta risk = 0.2 in a bilateral contrast) [18]. Calculations were performed using GRANMO v7.12 software [19]. Effect estimates of intracluster correlation coefficient in cluster randomized trials in primary care were generally lower than 0.05 [20]. The effect of the design corresponded to 1.7. Assuming these values, the final calculated sample size was 167 participants in each group (*n* = 334 overall).

## 4. Recruitment and Allocation

### 4.1. Randomization of Therapists

The investigators considered to randomize each child for allocation either in the intervention or control groups. However, children had a pediatrician and a nurse already assigned for regular health control. If all the pediatricians and nurses were responsible to perform motivational and control interventions, there was a high risk of contamination of the control intervention by motivational techniques. Therefore, to ensure no interference between the control and the intervention groups, and to avoid differences by socio-economical levels according to the location of centers, we performed randomization by clusters, where each primary care center was a cluster. In each primary care center, pairs of pediatricians and pediatric nurses worked as a team and were called a basic care unit (BCU), and this was considered as the unit of randomization. So, each team of pediatrician and nurse was randomized to be part of either the intervention or the control groups. Eight primary care centers were recruited in the first round (February 2016) which included 62 pediatricians and pediatric nurses. This means that the primary care centers had half of the BCU as control therapists and the other half as intervention therapists, when possible. In a second round, in January 2017, 7 additional primary care centers and their 20 pediatrician–pediatric nurse pairs were recruited and randomized by clusters to participate as a control or intervention BCU. Finally, there were a total of 82 pediatricians and pediatric nurses from 15 centers, distributed in 44 BCU taking part in the study as therapists (21 in the control group and 23 in the intervention group). Given the nature of the intervention, participants or researchers could not be blinded. Randomization was performed 1:1 with the EPIDAT 3.0 statistical Program [21].

### 4.2. Training of Therapists

All of the pediatricians and pediatric nurses received a 4-h training course during which they received information on the rationale and design of the project, good clinical practices and methods to assess the obese participants. 

After this, only the intervention group therapists followed with the training on the Obemat2.0 motivational intervention, which consisted on 12 h of additional training (two sessions of six hours). 

**Figure 1:**
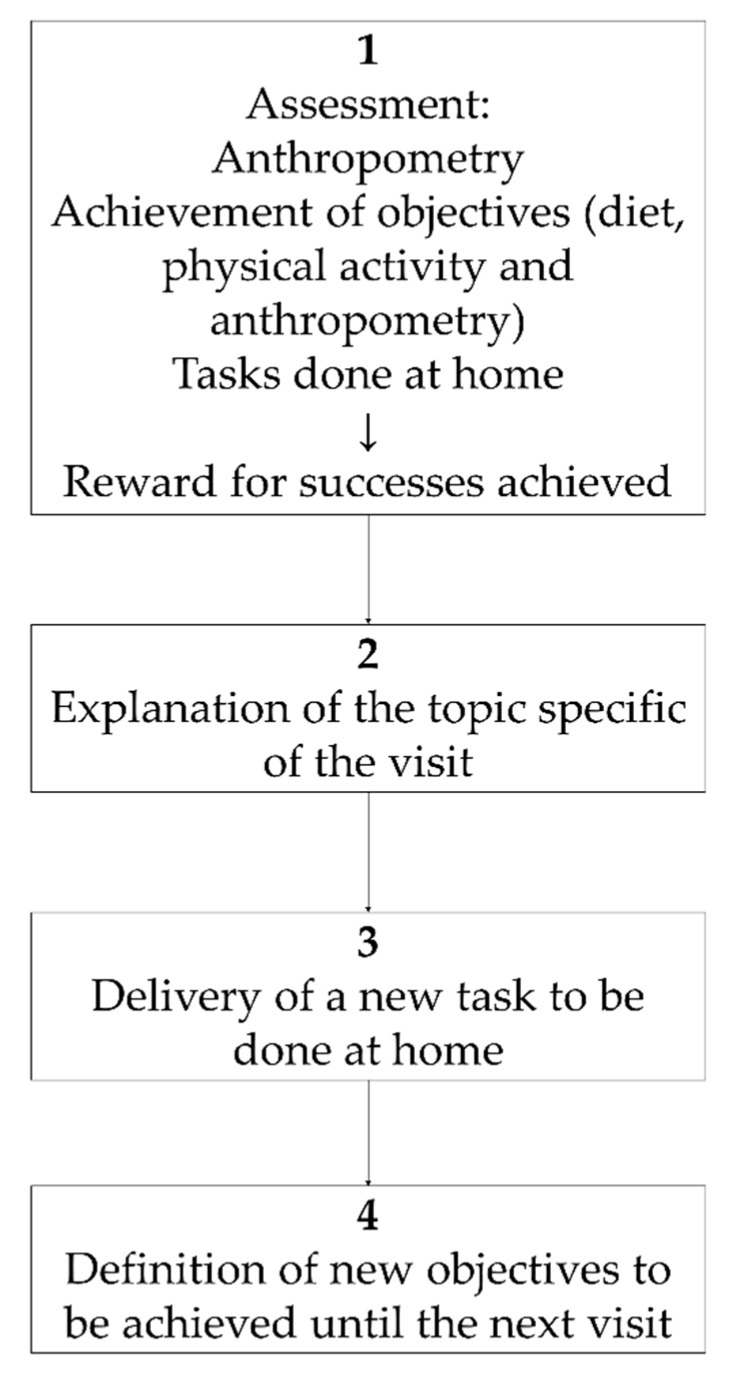
Obemat2.0 motivational interview structure within each visit.

The first of these two dedicated sessions consisted of: 1h for theory of the motivational approach, the basis to apply it to childhood obesity treatment, and how to structure the interview (Figure 1). After this, the training consisted of a detailed explanation of the activities to be carried out at each visit during the motivation-based interviews with the patients (Table 1). This session was dedicated to the motivational interview ending with a role-play activity in which therapists acted as such, or as parents or as children with obesity during a motivational interview.

The last of the training sessions focused on the three workshops in which the children in the intervention group would take part: (1) Motivation to increase physical activity, (2) labeling of food products and recommended portions, and (3) cooking healthy recipes. 

## 5. Participants

Inclusion criteria: Age range between 8 and 13 years at enrolment and a BMI > 97th percentile of the Hernandez references from 1988 [22] as indicated by the Guidelines for Clinical Practice on the Prevention and Treatment of Childhood and Adolescent Obesity of the Spanish Health System [23] for childhood obesity diagnosis.

Exclusion criteria: Children with eating disorders, families not available to attend scheduled visits, simultaneous participation in another clinical trial, presence of endocrine disorders (growth hormone disorder, hypothyroidism, Cushing’s disease, precocious puberty or other) and lack of command of local languages.

## 6. Recruitment and Follow-Up of Participants

The primary care centers of the study region are organized in BCUs, so that each BCU provide public health care to an assigned part of the population of the area. Therefore, pediatricians and/or pediatric nurses recruited children during their regular clinical practise at primary health centers, and the children with obesity enrolled in the study belonged to the control or intervention group depending on the BCU to which they belonged.

**Figure 2:**
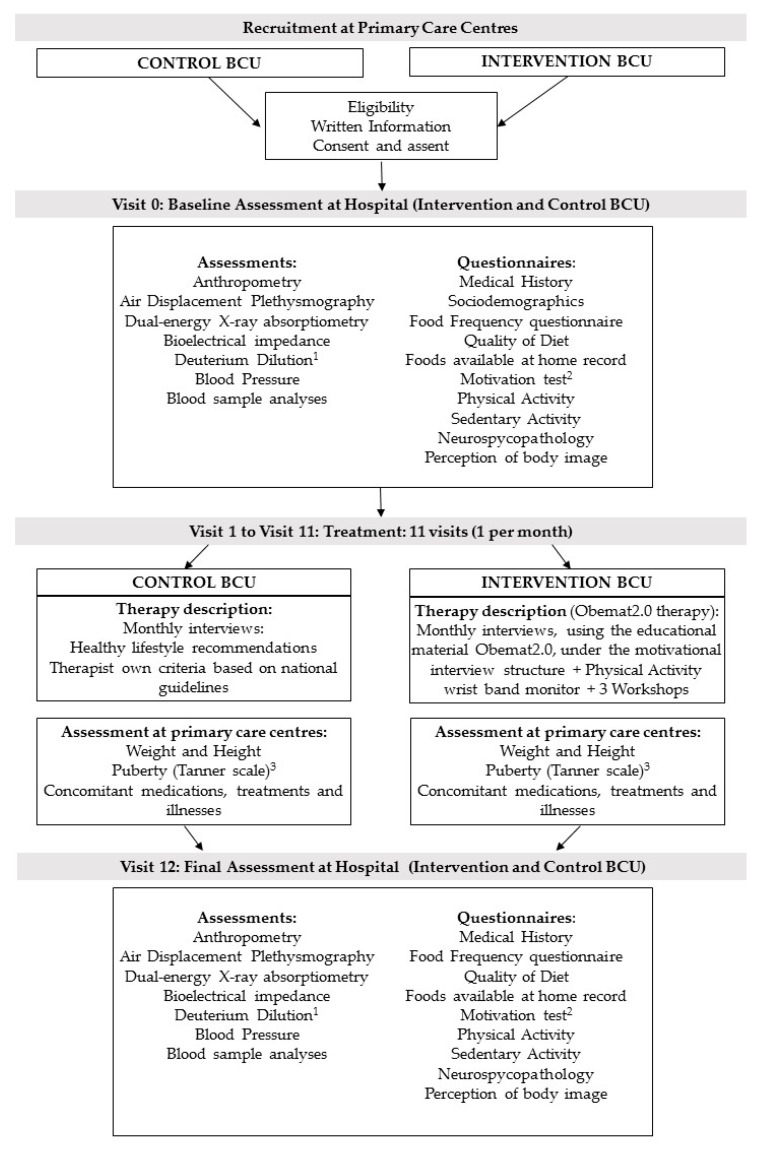
Flow diagram of recruitment, assessment per visit, and intervention. BCU: Basic Care Units; ^1^ Deuterium dilution analysis in a subsample; ^2^ Motivation test only in the intervention group; ^3^ anthropometry at primary care centers was performed on a monthly basis, except for the puberty development, that was assessed only at visit 1 and 11.

After checking the eligibility criteria, therapists gave written information to the parents or caregivers and adapted information to the children (if they were 12 years or older). When they agreed to take part, they gave the signed consent (parents) and assent (children aged 12 years or older) and were enrolled in the study. Baseline assessment took place at the reference Hospitals (Hospital Universitari de Tarragona Joan XXIII and Hospital Universitari Sant Joan de Reus) and included anthropometry, blood sample analyses, measures of body composition (i.e., fat mass, fat free mass, total body water, bone mineral content) using appropriate techniques as dual-energy X-ray absorptiometry (DXA), air displacement plethysmography (Bod Pod^®^), bioelectrical impedance, deuterium dilution (in a subsample), and blood pressure. A sociodemographic questionnaire, medical history, index of diet quality (KIDMED) [24], and a food frequency questionnaire [25] were completed during the interview. Furthermore, a physical activity questionnaire (PAQ-C) [26], sedentary activity questionnaire (ASAQ) [27], a neuropsychological test, a perception of body image test, and a form designed to record all foods present at home were given to the patient and his/her family to be completed at home and returned at the next visit. Furthermore, the intervention group completed a short motivation test (Figure 2). 

After the baseline assessment at the reference hospital, the participants started the treatment which was conducted by their regular pediatrician and/ or pediatric nurse at the primary care center, where they were visited monthly (11 visits in total). At all visits, children were weighed and height was measured. In addition, at visits 1 and 11, the pediatrician assessed pubertal development status (according to the Tanner Pubertal stages [28,29]), whether they showed acanthosis nigricans and/or there were any pathological result emerging from blood sample analyses. 

After visit 11 was performed, children were again assessed at the reference hospital (visit 12) as done at the baseline visit (visit 0) (Figure 2).

The children nor their families in the intervention or the control groups received any incentive nor benefit or payment because of their participation in the study. On the other side, participants did not have to pay for the treatment or visits.

## 7. Protocol Variation

Although the overall participation of the children with obesity and their families was planned to last 12 months, delays (considered as normal within the clinical practise) lead to a variation of the protocol. Reasons for delay included changes in appointments for children’s exams, family vacations or family problems, or delays caused by professionals’ illnesses or vacations. With this protocol variation, children’s follow-ups and final assessments could take place during up to 15 months.

## 8. Description of the Interventions

The treatment of both groups (control and intervention) took place in the primary care services by their usual pediatrician and/or pediatric nurse, along with the 11 monthly visits, each visit lasted approximately 20 min.

### 8.1. Usual Care (Control Group)

Children assigned to the control group received advice as recommended by the Guidelines for Clinical Practice on the Prevention and Treatment of Childhood and Adolescent Obesity of the Spanish Health System [23]. At each visit, the family received explanations about carrying out a balanced diet, divided into five meals, in order to provide a moderate energy intake reduction (assessed qualitatively, recommending exemption of energy-dense superfluous foods, restricting eating out of meals and reducing the size of the portions, if this was necessary). Specific dietary recommendations were four to five portions of fruits and vegetables per day, increasing wholemeal cereal products, avoidance of sugared beverages, cakes and pastries, junk food, fried food products, energy dense dairy desserts, and oil-based sauces. The pediatricians and/or nurses recommended preparing the same meals for the whole family and limiting the access of the child to energy dense food products. Therapists recommended an increase in physical activity, both in terms of leisure activity, and regular sports engagement. At monthly visits, therapists also measured weight and height, and assessed whether the family followed the recommendations. The pediatricians and/or nurses in the control group did not receive specific instruction to promote family tasks such as recoding food intake or physical activity; however, the therapists asked about these topics during the interviews in a non-structured way with the objective to reinforce compliance with recommendations.

### 8.2. Obemat2.0 Intervention

The intervention group followed the motivational interviewing schema described in Figure 1 during the 11 visits, took part in three workshops and participants got a wristband physical activity monitor (Figure 2). To guarantee the follow up of the motivation interview schema (Figure 1), printed material was designed and provided to the intervention BCU. That printed educational material was used with each child at every visit. It contained a section to ensure the revision of goals achievement, a section to explain the specific topic for each visit, a document to execute the task to be done at home and a last section where the agreed objectives to be accomplished until the next visit were recorded. Table 1 shows the details of topics and tasks planned at each single visit.

Furthermore, during the first four to six months, children in the intervention group could participate in workshops where therapists taught and/or trained and tried to motivate them on three different topics:

Workshop 1: Increasing physical activity by using an eHealth monitor (Fitbit^®^).

During the session, the children received a wrist physical activity monitor (Fitbit^®^), were taught about using it and were motivated to increase the physical activity records by doing leisure time family activities, walking, playing outdoors, etc. The monitor, connected to an App, allowed several features to fieldworkers. On one hand, children attending the workshop (maximum eight children per session) could be part of a Fitbit App^®^ group, so they were able to compete between them to increase their number of daily steps. Moreover, on the other hand, the fieldworker was able to download the time wearing the device and the daily steps of all participants to assess objectively their adherence to the treatment and the actual physical activity level. Each child in the intervention group received the wristband monitor by free.

Workshop 2: Food choices and balance.

The workshop had two different parts: at the beginning, children and their families received training to read labels of food products in order to discard energy dense food products.

During the second part, children and their families were trained to design a balanced diet and reduce portion sizes with the help of educational materials as plates and food models (from Portion Perfection (Great Ideas in Nutrition^©^, Tweed Heads, NSW, Australia). 

Workshop 3: Using healthy cooking methods. 

During the third workshop, children took part in a cooking session when they participated preparing healthy recipes.

In addition, at the end of the session, families received printed recommendations on preparing healthy foods, using low energy cooking methods. 

The reason to conduct the workshops (when possible) during the first four to six months of treatment was preventing the loss of interest that could be produced after several months of participation in a childhood obesity program. In other words, the aim of doing the workshops during the first six months was contributing to the child’s motivation.

Online Supplementary Table S1 provides the Template for Intervention Description and Replication checklist.

## 9. Outcome Measures

Children from both study groups were invited to take part in the same assessments at baseline and the end of the intervention (visits 0 and 12, except for vascular and the respiratory function tests, which were only assessed at the end) at one of the two reference Hospitals (but by the same research team). Between the two main visits, weight and height were recorded monthly, and other medical history aspects (described below) at visit 1 and visit 11 at the primary care centers as well.

### 9.1. Primary Outcome

The primary outcome was change in BMI z score between visit 0 and visit 12.

### 9.2. Secondary Outcomes

Secondary outcomes were changes in body composition (fat mass index, fat free mass index), systolic and diastolic blood pressure z scores, triglycerides mg/dL, HOMA-IR index and low density lipoproteins (mg/dL) between visits 0 and 12 (See the extended list of secondary outcomes in Table 2).

## 10. Outcomes and Variables

The Table 2 shows a summary of tests and measurements performed per visit.

### 10.1. Anthropometry

Trained study personnel measured weight (kg), height (m), and waist circumference (cm) at baseline and final visits using a SECA769 scale (precision 50 g), SECA 216 Stadiometer (precision 1 mm) and Holtain waist circumference non-extensible tape (precision 1 mm). In addition, pediatricians or pediatric nurses at primary health care centers measured weight and height at monthly visits. Waist circumference was measured at three different levels: at the iliac crest, at the mid-point between the iliac crest and the lower rib, and encompassing the maximum circumference. body mass index (BMI) (kg/m^2^) was calculated as $BMI\;\left(\mathrm{kg}/m^{2}\right)=\frac{weight\;\left[\mathrm{kg}\right]}{height\;{\left(m\right)}^{2}}$, and the z score of the BMI (BMI z score) for age and gender according to the World Health Organization (WHO) references [30] was calculated using the WHO software [31].

### 10.2. Body Composition

Body composition was assessed by means of several methods to obtain the gold standard measures of each component of the named “four components model” [32] at the beginning and the end of the treatment, always in fasted conditions.

Dual-energy X-ray absorptiometry (DXA): Bone mineral content (g) and density (g/cm^3^) were obtained by means of Axial Lunar Prodigy Full Advance device and the Software EnCore 2014 v15.20.002 (GE Lunar Corporation, Madison, WI, USA). The patient lay in a supine position, in underwear without any metallic object, and the toes of the two feet set one beside the other. Output variables were bone mineral content (g), bone mineral density (g/cm^3^), fat mass (kg) and fat free mass (kg) for the whole body, trunk and limbs. 

Air displacement plethysmography (Bod Pod^®^): the child was assessed in duplicate by Bodpod instrumentation (Life Measurements, Concord, CA, USA) wearing tight-fitting underclothes or swimsuit and a swimming cap to discard the air existing between hairs. Results were the mean of two or three measures of body volume of 50 s lasting each one. If the two first measures were consistent (differences between measures <150 mL of body volume), a third measure was not necessary. Thoracic gas volume was predicted by the BodPod during normal tidal breathing [33] and subtracted from total body volume in subsequent calculations.

Based on the principles of densitometry, body density was calculated by following the general equation of densitometry

$$Body\;Density\;\left(\frac{\mathrm{kg}}{L}\right)=\;\frac{Body\;Weight\;\left(\mathrm{kg}\right)}{Body\;Volum\;\left(L\right)}$$

and then this body density was used to predict *Fat Mass* (%) from Lohman’s equation [34]:

$$Fat\;Mass\;\left(\%\right)=\frac{C1}{Body\;Density-C2}\times 100$$

where *C1* and *C2* are constants based on age and gender.

Fat Mass was converted to kg

$$Fat\;Mass\;\left(\mathrm{kg}\right)=\frac{Fat\;Mass\;\left(\%\right)\times 100}{Body\;weight\;\left(\mathrm{kg}\right)}$$

Then, *Fat Free Mass* (kg) was obtained from subtracting *Fat Mass* (kg) from total *Body Weight* (kg).

Fat Free Mass (kg)=Body weight (kg)− Fat Mass (kg)

Deuterium dilution: deuterium dilution was used in a subsample (*n* = 75) as the gold standard measure for total body water (kg) determination [35]. This assessment was not performed in the overall sample because of budget constraints. Each subject provided a baseline urine sample prior to receiving a weighed oral dose of ^2^H_2_O. The dose was the equivalent of 70 mg·kg^−1^ body weight ^2^H_2_O. Parents got sample collection forms, written instructions (that trained study personnel delivered and explained) and the necessary material for urine collection. Instructions stated to collect samples of the child’s urine for the following five days after dosing, at a similar time of day provided it was not the first void of the day, and to preserve them refrigerated. Urine samples were kept frozen until being shipped frozen to MRC Elsie Widdowson Laboratory, Cambridge, UK. For ^2^H enrichment, samples of 0.4 mL were placed in 3.7 mL glass vials and flush-filled with hydrogen gas, and then equilibrated for six hours in the presence of a platinum catalyst. The headspace of the samples were then analyzed using a continuous flow IRMS (Sercon ABCA-Hydra 20-22, Sercon Ltd, Crewe, UK). All measurements were made relative to V-SMOW (Vienna Standard Mean Ocean Water) using calibrated laboratory standards. Analytical precisions (SD) are better than ± 1.3 ppm for ^2^H. TBW was calculated using the zero-time intercept of ^2^H turnover and corrected for non-aqueous exchange within the body. Hydration factors [36] were used to derive fat free mass (FFM) (kg). Fat mass (FM) (kg) was calculated as the difference between body weight and FFM.

Bioimpedance: Total bioelectrical impedance (BIA) with a high-frequency constant current (50 KHz, 500 µA) was assessed in duplicate with the 8-electrode BIA using the Tanita BC-418 (Tanita corporation, Tokyo, Japan). The child stepped on the scale barefoot and dressed in underwear, and instructed to find the right position to get in contact with the foot electrodes. The palms of the hands were on top of the device handles, with the fingers touching the lower electrodes and the thumbs placed straight along the electrode at the top. Outputs were impedance (Ω), total body water (TBW) (kg), FFM (kg), and FM (kg) for the whole body and the segments (trunk and limbs).

As body composition changes with age as part of human development, all body composition variables were standardized as z scores according to age and gender [37].

Blood sampling: at baseline and final visits, a trained nurse extracted a blood sample from the child’s in fasting conditions. Glucose (mg/dL), insulin (mIU), low density lipoproteins cholesterol (LDL) (mg/dL), high density lipoproteins cholesterol (HDL) (mg/dL), total cholesterol (mg/dL), triglycerides (mg/dL), liver enzymes (Alanine trasnsaminase, Gamma-Glutamyl Transferase, Aspartate transaminase), thyroid hormone (TSH), blood cell count, iron, ferritin, transferrin, creatinine, urea, C-reactive protein, and vitamin D (mg/dL). Insulin resistance index (HOMA-IR) was calculated as

$$HOMA-IR=\;\frac{Insulin\;\left[\frac{\mathrm{uU}}{\mathrm{mL}}\right]\times Glucose\;\left[\mathrm{mmol}/l\right]}{22.5}$$

Medical history: health records were consulted to obtain birth characteristics (delivery type, gestational age and birth weight, length and head circumference) and feeding type in early life (duration of breastfeeding and complementary feeding introduction).

Sociodemographic questionnaire: at the baseline interview, the study personnel performed the sociodemographic questionnaire including information on gender, date of birth, household income, level of education, occupation and employment of father and mother.

Medical examination: pediatricians or nurses explored Tanner maturation stage [28,29] and presence and placement of Acantosis Nigricans at visit 1 and 11. Trained study personnel measured systolic and diastolic blood pressure (mmHg) at baseline and final visit (at least 20 min after arriving to the study center) in duplicate (with a time slot of 5 min between measures ) using a Dinamap Pro 100 device on the left arm, while the child remained sat down with the arm laying comfortably.

Diet: diet was assessed qualitatively by means of a short Food Frequency Questionnaire validated in a similar population [25] and a questionnaire to assess the adherence to Mediterranean Diet in children (Kidmed) [24] completed by parents with the support of the children. Food items from the food frequency questionnaire were translated to grams using the same food portions of the validation study and the kidmed score was calculated. Finally, the participant families were asked to record all the foods available in the pantry and fridge at home at the beginning and the end of the study.

Physical and Sedentary Activity: parents with the help of their children completed validated questionnaires to assess physical activity [26] and sedentary activities [27] at the beginning and the end of the treatment.

Neuropsychology, behaviour, and self-perception: the Behavior Rating Inventory of Executive Functions (BRIEF) [38] was completed by parents to get a score on the inhibitory control, shift and emotional control. The Strengths & Difficulties questionnaire (SDQ) [39] was completed by parents as well to obtain scores for emotional symptoms, conduct problems, hyperactivity/ inattention, peer-relation problems, pro-social behavior, and “total behavior difficulties score”. Parents’ [40] and child’s self-perception of body figure and parents’ perception of the child’s figure were assessed by means of printed figures [41]. Misperception of body figure was assessed by the difference between perceived BMI and actual BMI; discomfort with body figure was assessed by the difference between perceived and desired images.

Motivational interview: the children in the intervention group (at the beginning and the end of the treatment) completed a questionnaire in which they should state several personal thoughts as: (1) the degree of willingness to lose weight, (2) the reason why they would like to lose weight, (3) which positive things they would get from becoming thinner, and (4) the negative aspects of following the dietary recommendations and physical activity advice.

### 10.3. Vascular Function

To measure carotid intima media thickness (cIMT), we used a My Lab 50 X-Vision sonographer (Esaote SpS, Genova, Italy) with a linear array ultrasound probe small parts broadband transducer (5-12MHz). We identified the far wall of the common carotid artery (1cm proximal to the bifurcation), the bifurcation, and the internal carotid artery pf the left and right carotid arteries. Measurements of cIMT were performed in vivo at the predefined points using QIMT© radiofrequency image processing software (Esaote SpA, Genova, Italy). We defined pathological cIMT as the 75th percentile of cIMT values in the general population banded with respect to age and sex, and plaque as a focal structure encroaching into the arterial lumen by at least 0.5 mm or 50% of the surrounding IMT value, or a thickness >1.5 mm.

Arterial stiffness expressed by the pulse wave velocity (m/s), carotid distensibility (μm), and augmentation index (%) was measured directly at both common carotid arteries using the ultrasound linear probe (5-12 MHz) as a tonometer and analyzed in vivo by the QAS^©^ radiofrequency software (Esaote SpA, Genova, Italy). Maximum and minimum carotid diameters (μm) were acquired using the attained distension curves, and vascular stiffness parameters were calculated after calibration for blood pressure. Carotid distensibility was the change in diameter of the carotid artery secondary to intravascular volume expansion caused by the left ventricle systole. The pulse wave velocity was obtained from brachial blood pressure and the accurate measurements of diameter and change in diameter of carotid arteries. Augmentation index was measured by the pulse wave analyses and local pressure. To reduce observer variability, a single operator obtained and measured the images. Final values were the mean measurements of the right and left carotid arteries. 

### 10.4. Respiratory Function

With the aim to assess the degree of bronchial obstruction in these children with obesity, patients and their families were asked to complete the Easy Breathing Survey (EBS) test [42]. Children with a previous diagnosis of asthma or an EBS score ≥1 performed a forced spirometry and bronchodilation test. The spirometer used was a Sibelmed W20S BETA^®^ (Sibel S.A., Barcelona, Spain) and its software (511-BL0-MU1 Rev). Main outcome measures were forced vital capacity FVC (%), forced expiratory volume in one second (FEV_1_) (%), its ratio ($\frac{FEV1}{FVC}$) and Forced Expiratory Flux between the 25% and 75% of the FVC (FEF_25%–75%_). After the administration of a bronchodilator, FEV_1_ increment (FEV_1_Λ) was assessed [43]. All the measures were standardized as z scores according to the multi-ethnic reference values of the Global Lung Function Initiative and the European Respiratory Society [44] by means of the GLI2012^®^ desktop software [45].

### 10.5. Adherence to the Intervention

To assess the adherence to the therapy, investigators recorded overall attendance to visits and attrition rates in both the intervention and control groups, as secondary outcomes. Furthermore, within the motivational program, investigators recorded the attendance to group sessions and the time using the wearable physical activity monitor (minutes/ day, days/ month) to assess the efficacy of the different parts of the intervention.

## 11. Conditions for Discontinuation of Participation in this Study and Follow-Up Actions

Conditions for discontinuation of participation in this study were illnesses diagnosis that could bias the study (such as neuropsychological, endocrine diseases considered as exclusion criteria) or that could affect the participant’s safety, such as eating disorders (for example, a pathological behavior to lose weight). 

## 12. Follow-Up of Adverse Events

This study consisted of the implementation of a motivational program to promote behavioral changes for a healthier lifestyle. The professionals providing the advice are registered pediatricians, nurses and registered dieticians working on pediatric clinics. There is little likelihood of any health hazards. If any serious adverse event occur, it would be reported in line with the Consort guidelines [46]. Until now, no hazards have been reported nor identified.

## 13. Statistical Analysis Plan

The statistical plan was testing the effect of the motivational intervention through a two-way ANOVA model to find differences in BMI z score increments (baseline to final visit) between groups (control vs. intervention); we considered adjustment by possible confounders such as gender, age diet, physical and sedentary activity, feeding in early life and nutritional status at birth.

The statistical plan also included similar models to test the secondary outcomes such increments (baseline to final visit) in systolic blood pressure, diastolic blood pressure, FM and FFM from a four-component model, triglycerides, LDL, HOMA-IR, and psychosocial problems. Specific models to assess the factors to adhere to the treatment were planned as well. These models had as dependent variables attendance (number) to interviews, and included the intervention vs. control group as a main factor; parents’ BMI, parents’ education level, baseline perception of body image; gender and age were possible modulators. The statistical plan was included as well the analysis of the extent in which the different motivational tools could influence BMI z score, by using as independent variables the attendance to individual interviews, to group sessions and the use of the physical activity monitor.

## 14. Ethics

The study followed the rules of the Declaration of Helsinki [47]. The ethical committees holding the activity of all the involved study centers approved the protocol: CEIC Hospital Universitari de Tarragona Joan XXIII (2 March 2016, code CP.OBEMAT2.0-C.I.01p/2016), CEIC Hospital Universitari Sant Joan de Reus (29 January 2016, code 16-01-28/1ass2), CEIC IDIAP Jordi Gol (26 November 2015, code PI14/116). If any amendments of the protocol were made, the Ethics Committees were notified as necessary. All parents or legal guardians signed informed consent prior to study enrollment. Children aged 12 years or above signed informed assent to participate in the study as well. 

## 15. Study Status

The recruitment started (first patient-first visit) in June 2016. The fieldwork is expected to end in June 2019. 

## 16. Discussion

The study will assess whether a multicomponent motivational program, including a bundle of motivational strategies (such as motivational interview supported by educational materials, a wearable physical activity monitor and group sessions) conducted in primary centers by therapists with 12 h of specific training could be more effective than usual care.

The motivational interview relies on the basis that the child and the family should feel by themselves the motivation to improve their lifestyle, and to achieve that, clinicians should adopt a consultative role through which families have the responsibility to be active in their care [48]. The clinician is encouraged to have a non-judgmental position, including understanding resistance to change and trying to drive the patient to resolve ambivalences [49]. The motivational interview as way of inducing to changes in health care settings has been the focus of recent reviews providing insights into its usefulness [50]. 

To our knowledge, motivational-based interventions to lose weight have been conducted in young adults, with positive effects on weight control, cardiovascular risk factors and self-esteem, as reported by a systematic review published in 2009 by Poobalan et al. [6]. 

In children, a review published in 2015, found three studies reporting on positive effects of motivational interview on BMI and other obesity-related behavior outcomes [51]. The authors concluded that motivational interviewing might be applicable in childhood obesity, but there was lack of research on this specific sample. A more recent randomized clinical trial on motivational interview counselling to treat childhood obesity concluded that therapists providing motivational interview and registered dieticians were able to achieve significantly greater BMI reductions compared to other health care providers and interventions [52]. The authors suggested that further, large-scale research was necessary, as is training of pediatricians and registered dieticians. The present clinical trial has provided a short period of training to a full set of pediatric professionals from primary care settings of a province. Therefore, the study will have the possibility to show whether this kind of training was effective in improving childhood obesity treatment at a wide scale.

There is a strong body of scientific literature supporting the use of dynamic groups to treat childhood obesity, as a method to make the children feeling engaged with their peers and the therapy, usually combined with personal interviews [12].

From the eHealth perspective, data supporting its use in childhood obesity is scarce [15]. In young adults, several works have reported no effect of wearable devices [14]. In children, the use of an electronic device could be considered more attractive and support self-monitoring to increase physical activity [13].

Among the barriers to treat childhood obesity, there might be some coming from the patient and his/her family (as lack of motivation to lose weight, non-healthy food preferences, etc.). However, some barriers may come from the health professionals. In a study assessing the self-efficacy of the pediatricians to treat childhood obesity, most pediatricians reported feeling ineffective in their ability to treat obesity and welcomed clinical resources for obesity management as practice-based tool kits [53]. One of the main strengths of the present study is that educational kits for children with obesity and their families have been designed; these kits will be available for use by health care providers once the intervention is finished.

## 17. Limitations and Strengths

The first limitation was that the nature of the intervention did not allow blinding the participants nor the therapists, which may affect the results. Furthermore, we acknowledge that randomizing the therapists but not the patients could be a limitation; and the recruitment of patients by the therapists could introduce certain degree of bias. Another possible limitation of this study is that given the combination of motivational strategies within the intervention program, we will not be able to determine which of the strategies (the motivational interview, the use of the eHealth device, the group sessions or the supporting educational materials) could have a more powerful effect on lifestyle changes and subsequent BMI improvement separately. Multivariate models will consider the adherence to the different strategies (i.e., attendance to visits and workshops) to assess, at least in part, its usefulness.

One of the most important strengths of this study is its randomized clinical trial design; to our knowledge, this is the first randomised trial to assess the effect of a structured motivational interview established in the primary care to promote behavior changes in children with obesity within this age range. 

Another relevant strength of the Obemat2.0 study is that the protocol was designed based on positive results obtained in a prior observational study using a similar intervention structure [11]. Furthermore, the homogeneity of the intervention was facilitated by providing printed educational materials to be given to families, specific for each visit (something that has previously been reported by pediatricians to be needed to overcome barriers in the treatment of childhood obesity [53]). A possible limitation of the study was that the actual fidelity of the therapists in the intervention group to follow the instructions could not be documented. However, we think that this could reinforce the robustness of the results, since this might reflect real clinical practise.

Last, but not least, one of the main interests of this study is that the intervention program did not focus on a specific highly trained clinic or team but has expanded to the full set of professionals in a multicenter primary care area. Establishing programs, which are feasible and can be widely spread in health systems as primary care, might be useful to arrive to the highest proportion of the obese population. This clinical trial should allow for testing the feasibility and efficacy of a program to treat children with obesity in primary care. Thus, a little improvement in a wide area would account for an overall great success.

In summary, we expect that this clinical trial could open a window of opportunity to support professionals at the primary care level to treat childhood obesity. 

**Obemat2.0 Study Group:** Closa-Monasterolo R, Feliu-Rovira A, Escribano J, Ferré N, Luque V, Zaragoza-Jordana M, Gispert-Llauradó M, Rubio-Torrents C, Gutiérrez-Marín D, Muñoz-Hernando J, Núñez-Roig M, Alcázar M, Sentís S, Esteve M (Pediatric Nutrition and Human Development Research Unit, Universitat Rovira i Virgili, IISPV, Reus). Monné-Gelonch R, Basora JM, Flores G, Hsu P, Rey-Reñones C, Alegret C (Unitat de Suport a la Recerca Tarragona-Reus, Fundació Institut Universitari per a la recerca a l’Atenció Primària de Salut Jordi Gol i Gurina (IDIAPJGol), Reus, Spain); Guillen N, Alegret-Basora C, Ferre R (Hospital Universitari Sant Joan de Reus); Arasa F (Hospital Verge de la Cinta de Tortosa, Institut Català de la Salut); Alejos AM, Diéguez M, Serrano MA, Mallafré M, González-Hidalgo R, Braviz L, Resa A, Palacios M, Sabaté A, Simón L (Hospital Lleuger de Cambrils, Sagessa); Losilla AC, De La Torre S, Rosell L, Adell N, Pérez C, Tudela-Valls C, Caro-Garduño R, Salvadó O, Pedraza A, Conchillo J, Morillo S (CAP Llibretat, Reus, Institut Català de la Salut); Garcia S, Mur EM, Paixà S, Tolós S, Martín R, Aguado FJ, Cabedo JL, Quezada LG (CAP Marià Fortuny, Reus, Sagessa); Domingo M, Ortega M, Garcia RM, Romero O, Pérez M, Fernández M, Villalobos ME (CAP Salou, Institut Català de la Salut); Ricomà G, Capell E, Bosch M, Donado A, Sanchis FJ, Boix A, Goñi X, Castilla E, Pinedo MM, Supersaxco L, Ferré M, Contreras J (CAP Rambla Nova, Institut Català de la Salut, Tarragona); Sanz-Manrique N, Lara A, Rodríguez M, Pineda T, Segura S, Vidal S, Salvat M (CAP Les Borges del Camp-Montroig del Camp, Institut Català de la Salut); Mimbrero G, Albareda A, Guardia J, Gil S, Lopez M (CAP Sant Pere, Reus, Institut Català de la Salut); Ruiz-Escusol S, Gallardo S (CAP Bonavista-La Canonja, Tarragona, Institut Català de la Salut); Machado P, Bocanegra R (CAP Torreforta-La Granja, Tarragona, Institut Català de la Salut); Espejo T, Vendrell M (ABS Vandellòs-L’Hospitalet de l’Infant, Sagessa); Solé C, Urbano R, Vázquez MT, Fernández-Antuña L (CAP Muralles, Tarragona); Barrio M, Baudoin A, González N (CAP El Morell, Institut Català de la Salut); Olivé R, Lara RM, Dinu C, Vidal C (CAP Sant Pere i Sant Pau, Tarragona, Institut Català de la Salut); González S, Ruiz-Morcillo E, Ainsa ME, Vilalta P, Aranda B (CAP Sant Salvador, Tarragona, Institut Català de la Salut); Boada A, Balcells E (ABS Alt Camp Est, Vilarodona, Institut Català de la Salut); Michelle Venables, Priya Singh (MRC Elsie Widdowson Laboratory, Cambridge, UK).

## Figures and Tables

**Table 1:** Obemat2.0 intervention scheme.

Visit	Topic to Be Discussed	Task Proposed to Be Done
**V1**	General concept of obesity. Acknowledgement of the problem and self-willingness to change	The patient should complete a list with the pros and cons of following the treatment against obesity
**V2**	Recommendations for food shopping.	Parents should sign a compromise to follow the food shopping list recommendations
	Workshop 1: Strategies to increase physical activity by using an eHealth monitor	
**V3**	Dietetic balance Healthy Menu	To design a menu for the whole week which follows the healthy balance and is adapted to the family preferences
	Workshop 2: Food products labeling and recommended food portions	
**V4**	To explore daily physical activities that could be increased (such us walking to school, taking care of chores such as walking the dog)	To make a list of activities that the child could do to reduce sedentary behavior
	Workshop 3: Cooking methods. Workshop at the kitchen.	
**V5**	What can I do if I have “anxiety”? What to do, what kind of healthy snacks could I have?	To make a list of lifestyle behaviors the patient realize is doing properly and those that should be improved to treat his/her obesity
**V6**	Habits around the table: family meals at regular times without TV nor screens, avoiding conflicts about food during mealtime, table without any food not assigned to any member of the family (i.e., full piece of bread in the middle of the table, excess of cooked food available)	To set the rules and record the order and schedules of the family around meals
**V7**	Breakfast and mid-afternoon snacks	To keep a 7 day record of all breakfasts and mid-afternoon snacks eaten (the week prior to the next visit)
**V8**	Recommended portion sizes Preparing foods to avoid leftovers	To record during the next month how many days there is an excess of prepared food
**V9**	Different types of physical activity: sport, daily activities, daily displacements, family outdoors activities	To plan family physical activities such as biking, hiking, promenades, etc.
**V10**	To revise the recommended daily or weekly portions of the different food groups (fruits and vegetables 4–5 per day, pulses 2–3 per week, etc.). Distribution and balance in lunch and dinner within the same day	To do a 7 day food diary during the week prior to the next visit
**V11**	Food shopping: coming back to “avoiding the negative stimulus” and planning the shopping list Strategies to avoid the access to energy dense foods	Plan the family menu for the next week and plan the shopping list avoiding unnecessary energy dense foods

**Table 2:** Scheme of data collection per visit.

Parameter/Test	V0	V1	V2	V3	V4	V5	V6	V7	V8	V9	V10	V11	V12
Medical history: Birth characteristics, feeding in early life	∗												
Sociodemographic questionnaire	∗												
Medical examination: Tanner maturation stage, Acantosis Nigricans, systolic and diastolic Blood Pressure z score	∗	∗										∗	∗
Anthropometry: weight, height, body mass index z score	∗	∗	∗	∗	∗	∗	∗	∗	∗	∗	∗	∗	∗
Anthropometry: waist circumference	∗												∗
Body composition: Bone mineral content and density, Fat Mass, Fat Free Mass, Total Body Water (all standardized as z scores for age and gender)	∗												∗
Blood sampling: HOMA-IR, Lipid metabolism, Liver enzymes and Thyroid Hormones	∗												∗
Neuropsychology, behaviour, and self-perception: BRIEF score, SDQ score, perception of body figure	∗												∗
Diet: Food Frequency Questionnaire, Quality of Mediterranean Diet in children (Kidmed) and pantry	∗												∗
Physical and Sedentary Activity questionnaires, daily steps ^1^	∗												∗
Motivational interview ^1^	∗												∗
Adherence to treatment: attendance to visits, attendance to workshops ^1^, use of the eHealth monitor ^1^		∗	∗	∗	∗	∗	∗	∗	∗	∗	∗	∗	∗
Vascular function: Intima media thickness and vessels properties (ultrasound scan)													∗
Respiratory Function (Easy Breathing Survey, forced spirometry and bronchodilator test)													∗

^1^ Only in the intervention group.

## Supplementary Materials

The following are available online at http://www.mdpi.com/2072-6643/11/2/419/s1, Table S1: TIDieR-PHP items to appropriately report the study design.
