# Supplementary material for: The Obemat2.0 Study: A Clinical Trial of a Motivational Intervention for Childhood Obesity Treatment
Source: Nutrients. 2019 Feb 16;11(2):419. doi: 10.3390/nu11020419 (PMC6413236; doi:10.3390/nu11020419)
Supplement: Supplementary file 1 [file nutrients-11-00419-s001.pdf]

Supplementary table. TIDieR-PHP items to appropriately report the study design.

1 BRIEF NAME OF THE INTERVENTION: *Usual care (control group)* vs *Obemat2.0 Intervention (multicomponent motivational programme)*

2 WHY

The primary aim of the Obemat2.0 trial was to evaluate the efficacy of a multicomponent motivational programme for the treatment of childhood obesity, coordinated between primary care and hospital specialized services, compared to the usual intervention performed in primary care.

The rationale to conduct the proposed motivational intervention could be summarized in the following reasons:

- The lack of motivation is reported as one of the main barriers for the obesity treatment effectiveness
- The most significant weight reductions had been obtained by combining diet, exercise and motivational strategies
- Primary care health professionals have the closest contact to families and should offer motivational interviewing to achieve healthy lifestyle modifications

3 & 4 WHAT MATERIALS AND PROCEDURES

Treatment (both in the control and intervention groups): recommendations to the patient and family about carrying a healthy diet and increasing physical activity with the aim to improve Body Mass Index in obese children

The content of the core recommendations, both in the control and the intervention groups was similar and included the following general items:

- Performing a balanced diet including all food groups, divided in 5 meals per day
- Avoiding energy-dense superfluous foods and eating out of meals-time
- Reducing the size of the portions, if this was necessary
- Preparing the same meals for the whole family

Specific dietary recommendations were four to five portions of fruits and vegetables per day, increasing wholemeal cereal products, avoidance of sugared beverages, cakes and pastries, junk food, fried food products, energy dense dairy desserts and oil-based sauces. Therapists recommended an increase in physical activity, both in terms of leisure activity, and regular sports engagement

- *Control group:* Recommendations given to families were not standardized among therapists. This means that therapists explained at each visit what they felt it would be useful in a non-structured manner. This would reflect the actual recommendations given in usual primary care
- *Intervention group*: Recommendations given to families were standardized among therapists. This was achieved by means of 12h training and printed materials to be given to families at each single visit. Furthermore, the structure of the programme stablished tasks to be done by the families at home, to put in practice the recommendations. Specific topics and tasks proposed at each visit were:

Topics per visit	Tasks per visit
1. General concept of obesity. Acknowledgement of the problem and self-willingness to change	1. The patient to complete a list of the pros and cons of following the treatment against obesity
2. Recommendations for food shopping	2. Parents to sign a compromise to follow the food shopping list recommendations
3. Dietetic balance, healthy menu	3. To design a menu for the whole week which follows the healthy balance and is adapted to the family preferences
4. Exploring daily physical activities that could be increased	4. To make a list of activities that the child could do to reduce sedentary behaviour
5. What can I do if I have "anxiety"? What to do, what kind of healthy snacks could I have?	5. To make a list of lifestyle behaviours the patient realize is doing properly and those that should be improved to treat his/her obesity
6. Family habits around the table	6. To set the rules and record the order and schedules of the family around meals
7. Breakfast and mid-afternoon snacks	7. To perform a 7 days record of all breakfasts and mid-afternoon snacks eaten (the week prior to the next visit)
8. Recommended portion sizes. Preparing foods to avoid leftovers	8. To record during the next month how many days there is an excess of prepared food
9. Different types of physical activity: sport, daily activities, daily displacements, family outdoors activities	9. To plan family physical activities such as biking, hiking, promenades, etc.
10. To revise the recommended daily or weekly portions of the different food groups. Distribution and balance in lunch and dinner.	10. To do a 7 days food diary during the week prior to the next visit
11. Food shopping: coming back to "avoiding the negative stimulus" and planning the shopping list. Strategies to avoid the access to energy dense foods	11. Plan the family menu and the shopping list for the next week
Workshop 1: increasing physical activity by using a wristband monitor	
Workshop 2: reading the food product labels to discard energy dense foods and control of food portions	
Workshop 3: healthy cooking methods and recipes	

5 WHO PROVIDED<br>The therapy was designed by the Paediatrics, Nutrition and Human Development Research Unit at Universitat Rovira i Virgili, IISPV.<br>Children were recruited and treated by their assigned paediatrician and/or paediatric nurse at their usual primary care centre<br>Providers of the treatment, both in the control and intervention groups were paediatricians and nurses from primary care centres of the Tarragona area.<br>Workshops on physical activity were carried out by a physician trained on using the wristband monitor, responsible of creating the physical activity competition groups in the app.<br>Workshop on labelling products and control of food portions were carried out by registered dieticians<br>Workshop on healthy cooking methods and recipes were carried out by a registered dietician and a cook
6 HOW<br>• *Control group:* Recommendations given to families by means of 11 personal interviews. Therapists in the control group received 4h basic training about good clinical practices and recommendations to carry out a healthy diet and increasing physical activity as stated in National Guidelines<br>• *Intervention group*: Recommendations given to families by means of 11 personal interviews (according to the received training and having specific printed), three group sessions and use of an eHealth device to monitor physical activity. Therapists in the intervention group received 4h basic training about good clinical practices and recommendations to carry out a healthy diet and increasing physical activity as stated in National Guidelines, plus 12h training on motivational interview and the content of the visits and educational materials
7 WHERE<br>Geographical scope: regional<br>Setting:<br>• Baseline and final assessment was performed at the reference hospitals by nutrition researchers<br>• Interventions provided by paediatricians and nurses at primary care centres. The health centre at which the treatment was provided was the regular centres assigned to families by living area
8 WHEN AND HOW OFTEN<br>Baseline assessment at the reference hospitals<br>After this, there were 11 personal interviews at primary care centres with a monthly frequency<br>Workshops (n= 3 and only in the intervention group), which were aimed to take place during the first 4 to 6 months<br>Final assessment at the reference hospitals<br>Overall duration of the study: 12 months (+3 months)
9 TAILORING<br>No tailoring specified
10 MODIFICATIONS OF THE INTERVENTION<br>The overall time per patient to conduct the assessment and intervention was expected to be 12 months. Due to delays of visits, inherent to the primary care (family vacations, child's exams, etc.), the research team decided to allow a total of 15 months to complete the study. There were no other modifications to the intervention
11 ADHERENCE STRATEGIES<br>Adherence actions: There were no payments or delivered benefits to participants.<br>No special adherence strategies were planned, as there was an underlying aim to mimic the feasibility of the programme in the primary care services. Furthermore, the adherence to the treatment was an outcome itself, since achieving a greater adherence in the intervention than in the control group would be a success itself.<br><br>Access to the treatment: patients should not pay any visit (nor in the control nor in the intervention groups)
